# Intradiscal Autologous Platelet-Rich Plasma Injection for Discogenic Low Back Pain: A Clinical Trial

**DOI:** 10.1155/2022/9563693

**Published:** 2022-10-10

**Authors:** Jianbo Zhang, Dongyang Liu, Qingjuan Gong, Jinsheng Chen, Li Wan

**Affiliations:** Department of Pain Management, The State Key Clinical Specialty for Pain Medicine, Guangzhou Medical University Second Affiliated Hospital, Guangzhou, China

## Abstract

**Background:**

Platelet-rich plasma (PRP) contains high concentrations of growth factors and cytokines and may promote healing and tissue formation and exert anti-inflammatory effects. PRP has been shown to improve intervertebral disc degeneration in vivo and in vitro. This study is aimed at evaluating the effectiveness of autologous PRP on discogenic low back pain (DLBP) at 48 weeks postinjection in patients who received a single intradiscal injection.

**Methods:**

All patients received a single intradiscal injection of PRP in a prospective trial. The pain scores, lumbar function, and adverse events were assessed at 1 week, 4 weeks, 8 weeks, 12 weeks, 24 weeks, and 48 weeks postinjection and compared to the preinjection values (0 weeks).

**Results:**

Data were analysed from 31 patients with a 94% follow-up rate. Compared to preinjection, pain and lumbar function were significantly improved, and there were significant differences (*P* < 0.05) over the 48-week follow-up. Twenty-two (71%) patients were classified as successes after the intradiscal injection of PRP. One patient received surgery at two weeks postinjection due to intervertebral discitis.

**Conclusions:**

Intradiscal injection of PRP can significantly relieve pain sensation and improve lumbar function in patients with DLBP over a 48-week follow-up period. Further randomized controlled clinical trials are needed to assess the effects of this injection therapy.

## 1. Introduction

Low back pain (LBP) is now regarded as a primary cause of adult disability, and as many as 84% of people have experienced back pain at least once in a lifetime [1]. In 2017, the Global Burden of Disease (GBD) showed that the incidence rate of restricted LBP activity was estimated to be approximately 7.3% [2]. With the increasing prevalence of low back pain, LBP has a significant impact on the societal economy. The Centers for Disease Control and Prevention of U.S. reported that approximately 70 million Americans currently suffer from LBP, and related expenses have been found to be up to $250 billion in wages and treatment costs each year—not to mention the loss of health over the years [3, 4]. This problem is not just a health service issue in the United States but a leading cause of disability worldwide [5].

Lumbar disc degeneration is the leading cause of discogenic low back pain (DLBP) [6]. The lumbar intervertebral disc (IVD) is composed of the annulus fibrosus (AF), nucleus pulposus (NP), cartilage, and bone endplates that connect the disc to the vertebrae [7]. There are basically no vascular tissues in the IVD, and the outer layer of the annulus is innervated by sinus vertebral nerves and sympathetic fibres [8–10].

Although the exact pathophysiological mechanism of IVD degeneration remains unclear, pathophysiological changes, including proteoglycan and type II collagen, typically progressively decrease, and dehydrated type I collagen increases, which results in tissue fibrosis [11, 12]. During the process of disc degeneration, proinflammatory cytokines, including IL-1 and tumour necrosis factor-alpha (TNF-*α*), are significantly increased, which disrupts the haemostasis of the extracellular matrix (ECM) of IVD tissues by increasing the production of matrix metalloproteinases (MMPs), nitric oxide, and aggrecanases [12–15]. Most patients with DLBP are significantly improved within 2 to 6 months of conservative treatment; however, 20% of patients experience recurring LBP [16].

The treatment of IVD degeneration includes surgical treatment and conservative treatment [17]. Conservative treatment includes rest, physical therapy, and anti-inflammatory drugs, with the main purpose of reducing the pain sensation of LBP. Surgical treatment of intervertebral fusion is aimed at reducing discogenic pain by eliminating motion between spinal segments. Recently, the use of autologous cells to treat degenerative disc diseases has become popular, including platelet-rich plasma (PRP), adipose mesenchymal stem cells, and bone marrow mesenchymal stem cells. PRP has been widely studied for the treatment of various musculoskeletal disorders; PRP contains higher concentrations of growth factors and is believed to promote healing and tissue formation [18]. Studies have shown that these growth factors are powerful substances that promote proliferation, cell migration, and synthesis of ECM proteins and collagen [19–21]. In addition, PRP has been found to have anti-inflammatory effects [22].

Preclinical studies have shown that concentrated PRP has a reparative effect on IVD cells [23]. A review article by Chang et al. concluded that PRP has a significant tissue repair effect to improve IVD degeneration in vivo and in vitro [23]. Several recent clinical trials have shown that PRP is safe and can effectively relieve low back pain and improve lumbar function in patients with DLBP [24–28]. However, there is little clinical evidence for the role of PRP therapy in the repair of IVD tissue, and clinical trials are needed to provide clinical evidence for the treatment of DLBP with PRP.

## 2. Methods

### 2.1. Study Design

This study was a prospective clinical study that mainly evaluated the effect of PRP therapy on the pain and function of patients with DLBP. The study was carried out from July 2019 to October 2020 and was approved by the Ethics Committee of the Hospital (2019-hs-22) and registered with the Chinese Clinical Trial Registry (ChiCTR1900024268). All patients participated in the trial after signing the informed consent form.

### 2.2. Participant Recruitment

**Figure 1:**
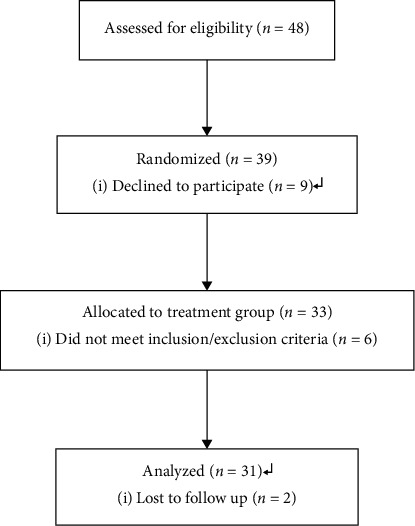
Flowchart of study participant enrollment, randomization, and analysis.

Forty-eight participants were eligible to participate in the clinical trial after evaluation at a single inpatient center between July 2019 and October 2021 according to the general inclusion and exclusion criteria (as shown in Table 1). A total of 9 participants declined to participate. A total of 39 participants who met the prediscography inclusion criteria were recruited into the trial. After discography, the results showed that 6 participants were excluded because of either the presence of grade V disc degeneration or lack of concordant pain at the time of injection with contrast (disc degeneration was defined by the disc degeneration grade/classification system) [29]. Finally, 33 patients participated in this trial, and 2 participants were lost to follow-up (Figure 1).

### 2.3. Diagnosis of Discogenic Low Back Pain

Participants who met general inclusion criteria with a history of chronic low back pain were enrolled in this trial after preliminary assessment. The presumed lumbar levels were preliminarily determined based on clinical symptoms and MRI positive findings of IVD degeneration, such as high-intensity zone (HIZ), disc protrusion, or endplate Modic changes [30–33]. However, it was necessary to exclude low back pain caused by lumbar facet joints or soft tissues.

Discography provoking concordant pain reproduction and disc block was performed for DLBP as reported in a previous study [34]. Meanwhile, discography requires a presumed disc and at least one negative control disc following ISIS guidelines for DLBP diagnosis [33]. A spinal puncture needle (22-gauge, 150 mm; Cosman, USA) was inserted into the nucleus pulposus in the center of the IVD under anteroposterior and lateral X-ray views. Then, intradiscal injection of radiocontrast agent (Omnipaque 240, Daiichi Sankyo, Japan) was used to provoke concordant pain. After concordant pain was triggered and assessed, disc block was performed with a 1 mL injection of 2% lidocaine (Heng-Rui, China). The degree of discogenic pain reduction was evaluated after 1 h and 24 h. The concordant pain of patients was induced by discography, and low back pain was relieved by disc block and then diagnosed as DLBP. After discography, the presumed lumbar level was scanned by computed tomography, and the morphological changes of the intervertebral disc were categorized according to the Dallas Discogram Classification [29].

### 2.4. PRP Preparation

PRP preparation was administered using a sterile preparation kit (Regen Laboratories SA Inc., Switzerland). Ten millilitres of whole blood was aspirated into a tube containing anticoagulant and via a Harvest Technologies Corporation (Plymouth, MA) centrifuge at room temperature (at 3,000 × *g* for 15 minutes) to produce 3-4 mL of PRP (buffy coat layer), which contained platelets, leukocyte-poor cells, and red blood cell-poor cells. The platelet concentration in the PRP was approximately three to five times higher than that in baseline whole blood. After preparation, 3 mL of supernatant (PRP) was aspirated into a sterile syringe for intradiscal injection.

### 2.5. Procedure for Injection of PRP

**Figure 2:**
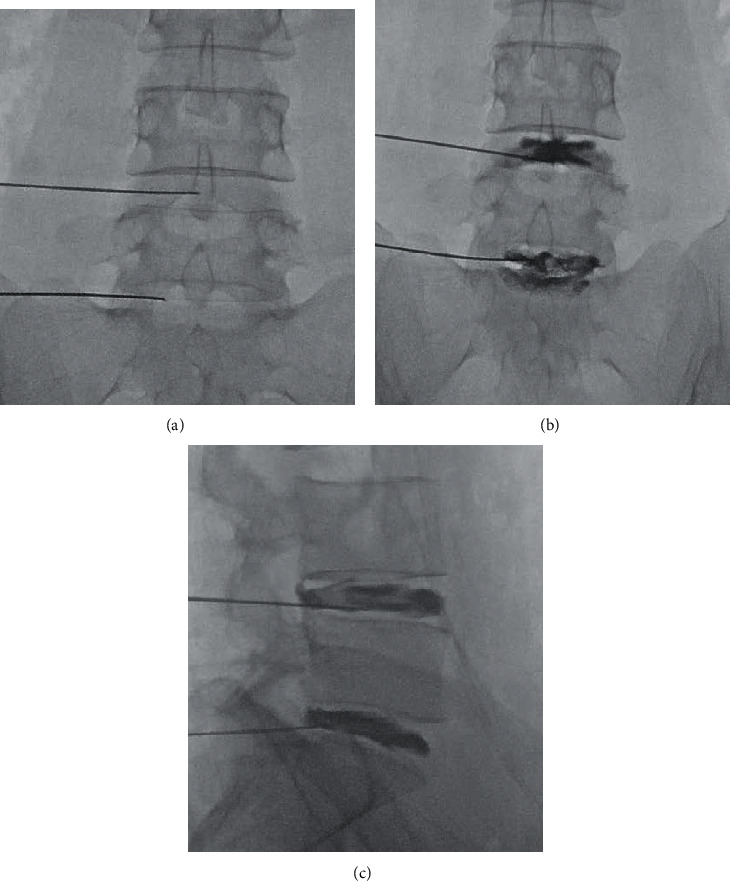
The spinal needle for platelet-rich plasma injection into the L4-L5 and L5-S1 discs under fluoroscopic guidance. (a) Anteroposterior view of needle puncture, (b) intervertebral disc imaging after injection of contrast agent in anteroposterior view, and (c) lateral view.

To prevent intraoperative infection, antibiotics were administered by peripheral venous access within 1 h before the PRP intradiscal injection. The participants were placed in a prone position on the X-ray operating table for the PRP injection procedure. The spinal needles were punctured to the presumed responsible disc under fluoroscopic guidance by anteroposterior (AP) and lateral extrapedicular discogram techniques. Before injection, the puncture site was sterilized and injected with a local anaesthetic of 0.5% lidocaine. A spinal needle (22-gauge, 150 mm) was inserted into the nucleus pulposus (NP) of the presumed pain-generating disc in the AP and lateral views under intermittent fluoroscopy (Figure 2). Approximately 2 mL of PRP preparation was slowly injected through a syringe at each disc level. If more than one disc had been reproduced with concordant pain, an equal dose of PRP preparation (approximately 2 mL) was injected into each of the presumed discs. After the injection of the PRP preparation, all participants were hospitalized to assess efficacy and adverse events for 3 days. Patients stayed in bed as much as possible during hospitalization. If there were no adverse complications, activities of daily living were permitted seven days after discharge from the hospital. Depending on the degree of pain relief, physical activity, such as exercise, was permitted at 1 month postinjection. To reduce the impact on pain assessment, only nonsteroidal anti-inflammatory drugs (NSAIDs) could be used except in the case of particularly severe pain (VAS scores of 8 points or more).

### 2.6. Baseline Measures

General demographic information of the participants was collected through medical records and questionnaires, including age, sex, number of levels, multiple levels injected, levels injected, and baseline scores. Baseline scores were obtained from participants before treatment and were assessed by the numeric rating scale (NRS), functional rating index (FRI), and 36-item short form health survey (SF-36) questionnaires.

### 2.7. Primary Outcome Measures

The SF-36 was scored in this trial, which only included the physical function (SF-36 physical function) and pain (SF-36 physical pain) sections. NRS pain scores consisting of current pain, best pain, and worst pain sections were used to assess pain. The NRS of pain is usually presented as a 100 mm horizontal line on which the intensity of the pain is indicated by a number between 0 (means “no pain at all”) and 10 (means “the worst pain imaginable”).

The functional rating index (FRI) was used to assess low back function. The FRI was a self-reporting instrument measuring the function of the participant's spine in motion and static state, including the performance of pain intensity, sleeping, personal, travel, and work.

The minimum clinically important difference (MCID) of the FRI, NRS, SF-36 physical function, and SF-36 pain scores was a change of 9 points, 2 points, 4.9 points, and 10 points [35–39], respectively. Patients were classified as “success” or “failure” based on their responses to the follow-up survey. “Success” was defined as meeting the MCID for both pain and function without the need for surgery. “Failure” was defined as either needing surgery or not meeting the MCID for pain or function.

### 2.8. Secondary Outcome Measures

Adverse effects of both treatments, such as increased pain, bleeding, infection, and complications such as nerve damage, were assessed. Any adverse events that occurred during the trial were recorded and assessed in relation to treatment.

### 2.9. Follow-Up Schedule and Its Measures

After informed consent was obtained, follow-up surveys including NRS, SF-36, and FRI were administered either online or by phone at 48 weeks following intradiscal PRP injections. The follow-up survey also included questions regarding side effect such as increased pain, infection, and nerve damage following the intradiscal PRP injections. Meanwhile, the patients were defined as “failure” or “success” according to the above MCID criteria. For patients who did not complete the follow-up survey, electronic medical records were reviewed to determine reasons for case dropout.

### 2.10. Statistical Analysis

Continuous variables are expressed as the means and standard deviations, while percentages and frequencies are indicated for discrete variables. The significant differences between preinjection and postinjection FRI, NRS, and SF-36 scores were analysed by 2-way repeated measures analysis of variance (ANOVA). Discrete variable data of successes and failures by intradiscal PRP injections were analysed by the *χ*^2^/Fisher exact test. All data were analysed by SPSS 20.0 (IBM Corp., NY, USA) with a significance level of *P* < 0.05.

A sample size of participants (27 treatment participants) was estimated by power analysis to achieve greater than 80% power to detect a 9-point change in FRI score with estimated standard deviations of plus or minus 15 in a 2-way repeated measures analysis of variance model with 7 time points.

## 3. Results

### 3.1. Patient Baseline Characteristics

Two participants were lost to follow-up; they were not included into the analysis. A total of 31 patients completed the follow-up survey at the time of preinjection (0 weeks) and at 1 week, 4 weeks, 8 weeks, 12 weeks, 24 weeks, and 48 weeks postinjection. The mean age at injection was 53.4 years (standard deviation: 8.5). Multiple levels were injected in almost half of the patients (*n* = 17; 55%). The current NRS pain score was 5.6 (standard deviation: 1.6). Baseline characteristics are shown in Table 2. Moreover, the Pfirrmann disc degeneration grade 3 was in 21 patients and grade 4 in 10 patients. All patients showed the HIZ sign of lumbar discs by MRI; however, only 11patients exhibited Modic changes but no one suffered acute symptom.

### 3.2. Quality Assessment of PRP and PRP Releasate

The mean platelet count of PRP was approximately 4.7 times greater than that of whole blood (whole blood [220.3 ± 51.6] × 10^9^ platelets/L; PRP [1025.3 ± 929.6] × 10^9^ platelets/L). The mean WBC count of PRP and whole blood were [7.20 ± 2.1] × 10^9^ cells/L and [0.20 ± 0.3] × 10^9^ cells/L, respectively. The average level of PDGF-BB in the PRP releasate was approximately 2.5 times higher than that in autologous serum (PDGF-BB (ng/*μ*L), autologous serum, 3.1 ± 1.6; PRP, 7.6 ± 3.3).

### 3.3. Pain

**Figure 3:**
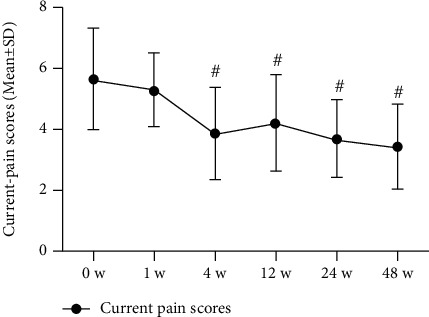
Current pain scores of NRS (*n* = 31). The changes in scores at baseline (0 weeks), 1 week, 4 weeks, 8 weeks, 12 weeks, 24 weeks, and 48 weeks. Statistical significance was analysed via 2-way repeated measures analysis of variance (ANOVA). This finding indicates no statistical significance for 1 week versus baseline (0 weeks); ^#^*P* < 0.001 versus baseline (0 weeks).

**Figure 4:**
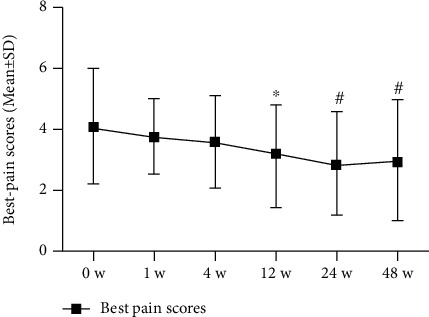
Best pain scores of NRS (*n* = 31). The changes in scores at baseline (0 weeks), 1 week, 4 weeks, 8 weeks, 12 weeks, 24 weeks, and 48 weeks. Statistical significance was analysed via 2-way repeated measures analysis of variance (ANOVA). This finding indicates no statistical significance for 1 week versus baseline (0 weeks); ^∗^*P* < 0.05 versus baseline (0 weeks); ^#^*P* < 0.001 versus baseline (0 weeks).

**Figure 5:**
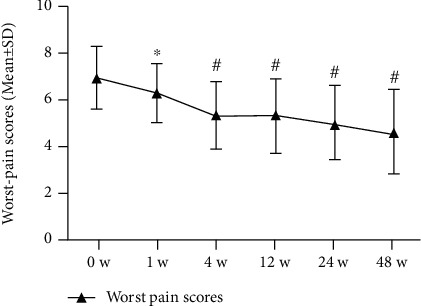
Worst pain scores of NRS (*n* = 31). The changes in scores at baseline (0 weeks), 1 week, 4 weeks, 8 weeks, 12 weeks, 24 weeks, and 48 weeks. Statistical significance was analysed via 2-way repeated measures analysis of variance (ANOVA). ^∗^*P* < 0.05 versus baseline (0 weeks); ^#^*P* < 0.001 versus baseline (0 weeks).

**Figure 6:**
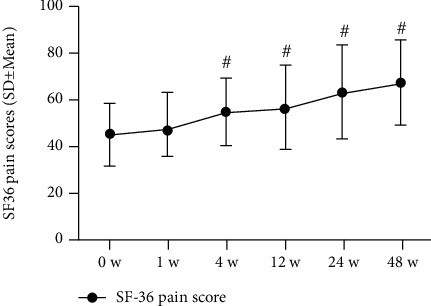
SF-36 pain scores (*n* = 31). The changes in scores at baseline (0 weeks), 1 week, 4 weeks, 8 weeks, 12 weeks, 24 weeks, and 48 weeks. Statistical significance was analysed via 2-way repeated measures analysis of variance (ANOVA). This finding indicates no statistical significance for 1 week versus baseline (0 weeks); ^#^*P* < 0.001 versus baseline (0 weeks).

Patients reported significant decreases in current, best, and worst NRS pain at 48 weeks postinjection as follows, respectively: 5.6 [1.6] to 3.4 [1.4], 4.1 [1.9] to 3.0 [1.9], and 6.9 [1.4] to 4.6 [1.8]; *P* < 0.001. SF-36 pain scale scores (higher = better) were also significantly improved (to 66.8 [18.1] from 45.0 [13.1] at 0 weeks; *P* < 0.001). All changes in pain scores were clinically significant, according to predefined MCID criteria (Figures 3–6; Table 3).

### 3.4. Physical Function

**Figure 7:**
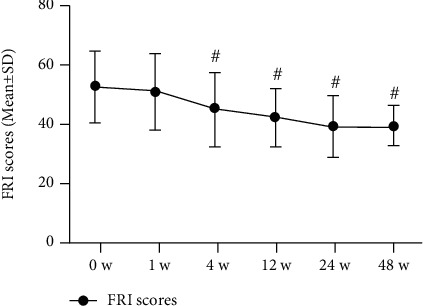
Functional rating index scores (*n* = 31). The changes in scores at baseline (0 weeks), 1 week, 4 weeks, 8 weeks, 12 weeks, 24 weeks, and 48 weeks. Statistical significance was analysed via 2-way repeated measures analysis of variance (ANOVA). This finding indicates no statistical significance for 1 week versus baseline (0 weeks); ^#^*P* < 0.001 versus baseline (0 weeks).

**Figure 8:**
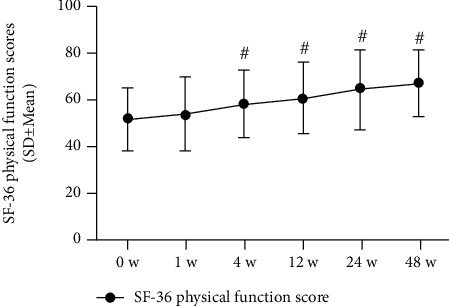
SF-36 physical function scores (*n* = 31). The changes in scores at baseline (0 weeks), 1 week, 4 weeks, 8 weeks, 12 weeks, 24 weeks, and 48 weeks. Statistical significance was analysed via 2-way repeated measures analysis of variance (ANOVA). This finding indicates no statistical significance for 1 week versus baseline (0 weeks); ^#^*P* < 0.001 versus baseline (0 weeks).

Significant improvements in function were observed at 48 weeks postinjection, as assessed by the SF-36 physical function scale (where higher scores indicate better function) and by the FRI (where lower scores indicate better function). SF-36 physical function scale scores improved from 51.8 (13.1) at baseline to 67.7 (14.4) at 48 weeks postinjection. The FRI scores improved from 52.5 (12.1) at 0 weeks to 39.2 (7.0) (*P* < 0.001). All changes in the clinical significance of the SF-36 physical function scores and FRI scores are shown in Figures 7 and 8 and in Table 3.

### 3.5. Pain Medication Usage, Additional Injections, and Physical Therapy

Of the 31 patients who completed the survey, 12 (38%) indicated that they had taken pain medication following the injection. Nine (29%) patients required additional physical therapy, and there were no patients who received additional PRP injections.

### 3.6. Success of Intradiscal PRP Injection

Successes and failures were determined using data from 31 patients. Of these, 22 (71%) patients reported clinically and statistically significant improvements in both pain and function at 48 weeks postinjection and were classified as successes in accordance with the MCID criteria. Nine (29%) patients were classified as experiencing failure because they did not meet the MCID criteria. One patient needed surgery after his intradiscal PRP injections and was classified as experiencing failure; this patient underwent surgery at 2 weeks postinjection due to intervertebral discitis associated with repeated needle puncture in the lumbar 3/4 disc. Sex and age at the time of injection had no effect on the success of the injection (Table 4); however, the number of segments and multiple levels injected had significant effects on the success of the injection (*P* < 0.05).

## 4. Discussion

Degeneration of the IVD is one of the leading causes of chronic DLBP in adults [40]. To assess the clinical efficacy of a single intradiscal autologous PRP injection in participants with chronic DLBP, this preliminary trial was designed in a prospective design study. The findings showed clinically and statistically significant improvements in pain and physical function at several time points compared to baseline.

The regeneration effect of PRP on IVD cells has been confirmed using in vitro and in vivo studies. An animal study reported that an injured IVD model treated with PRP immediately after injury resulted in the retainment of morphologic features, fewer inflammatory cytokines, and retarded IVD degeneration [41–43]. Furthermore, many clinical trials have demonstrated a significant reduction in pain and improvement in physical function of DLBP at different time points after treatment with PRP intradiscal injection. Of these clinical trials, a prospective, double-blind, randomized controlled trial demonstrated significant improvements in pain and function of DLBP patients with intradiscal PRP injection compared to contrast agent (control group) at a 1-year follow-up [25]. A prospective trial reported improvements in pain and function up to 6 months in 22 patients who underwent intradiscal PRP injection for DLBP [26]. Akeda et al. reported that intradiscal injection of PRP in patients with DLBP was a significant improvement in multiple assessments, including the Roland-Morris Disability Questionnaire (RDQ) and visual analogue scale (VAS), as well as X-ray and MRI (T2 quantification) during 6 months of follow-up [27]. Most recently, Cheng et al. reported that improvements in pain and function were sustained for much longer than 5–9 years of follow-up after intradiscal PRP treatment for DLBP [28]. This study suggested that intradiscal injection of PRP may have a long-term effect on repairing IVD degeneration.

In our study, the findings showed significant improvements in pain and function at the 48-week follow-up after a single intradiscal PRP injection, which is consistent with most of the above studies, in accordance with the MCID criteria of the NRS, FRI, and SF-36 [36, 38, 44]. Of these 31 patients with available follow-up data, approximately 71% (22 patients) of patients were classified as successes, and 29% (9 patients) of patients were classified as failures because they did not meet the MCID criteria. There were no effects of sex or age on the success of the injection. Age may be a vital factor in determining the ability of IVD cells to heal. Li et al. [42] reported an association between ageing and worsening of IVD degeneration. However, in our study, the findings suggested that age had no effect on the success of the injection, which may be related to the small sample size of this trial. In addition, the number of participants with multiple levels injected or the number of discs generating low back pain was relatively higher in the failure group than in the success group. This finding suggested that the increased number of levels injected and the number of lumbar segments are likely related to severe IVD degeneration, thus leading to more failed injections.

Importantly, discography provoking concordant low back pain was used only for the diagnosis of DLBP in this trial, which was similar to many previous studies [27, 28] and was also performed in accordance with ISIS guidelines [33]. The study reported that the injection volume of contrast agent for discography during this diagnostic procedure may reach 3 mL following the ISIS guidelines [33]. Therefore, insufficient residual volume may be available to accommodate an adequate PRP volume for treatment. Thus, in the present study, PRP was injected after discography for 3 days rather than immediately after discography, which is different from the results of a previous study [25].

Meanwhile, the safety and adverse events associated with this injection were also assessed throughout the whole follow-up period. In our study, 1 patient experienced severe low back pain after injection for half of a month. Based on the MRI scanning results, intervertebral discitis was observed at the injected disc level. The results may be related to repeated needle puncture, which might irritate the endplate immune infection responses. Fortunately, the patient showed significant improvement in pain and function after receiving surgery for discitis. In this case, repeated puncture was responsible for this adverse event, rather than PRP injection therapy, because PRP was promoting regeneration autologous substance, which is unlikely to have harmful effects on the disc [45, 46]. In summary, intradiscal injection of autologous PRP is safe and effective for treating patients with DLBP.

Although this study has demonstrated encouraging findings, there were several limitations in this trial. First, the sample size was relatively small, and a control group was not used. Without a control group, nonspecific treatment effects and natural recovery history cannot be ruled out, which may affect the interpretation of the effective results. Second, there is a lack of composition data of PRP on cell count, including platelets, red cells, and white blood cells, as well as biological analysis of various growth factors. Finally, there is no routine radiological assessment of the morphologic changes of the disc treated with PRP during follow-up, preferably with MRI analysis. Further clinical trials will be required to collect these data to confirm the effect of PRP injection on DLBP.

## 5. Conclusions

In summary, intradiscal injection of PRP is safe and feasible to treat patients with DLBP. Although our results are encouraging, more prospective, double-blinded, randomized, and controlled clinical trials would be required in a large sample size in the future, and the optimal composition and concentration of autologous PRP, whether multiple injections benefit or worsen outcomes, and what radiologic disc changes after injection at longer follow-up are all worth investigating.

## Figures and Tables

**Table 1:** Inclusion and exclusion criteria for study participation.

Inclusion criteria	Exclusion criteria
(i) Refractory discogenic source of low back pain persisting for ≧6 months (ii) Failure of conservative treatment measures (oral medications, physical therapy, and/or injection therapy) (iii) Maintained intervertebral disc height of at least 50% (iv) Disc protrusion less than 5 mm on magnetic resonance imaging or computed tomography scan (v) Concordant pain on discography, presence of a grade 3 or 4 annular fissure as determined by discography (vi) Absent alcohol or drug abuse within the past 5 years	(i) Presence of a known bleeding disorder (ii) Lumbar spine surgery within the past 6 months (iii) Pregnant or breastfeeding (iv) Active infection (v) Allergy to contrast agent (vi) Severe psychological illness (vii) Severe spinal canal compromise at the levels to be investigated (viii) Extrusions or sequestered disc fragments (ix) Spondylolysis (x) Inflammatory arthritis (xi) Negative provocation discography (xii) Presence of a grade 5 annular fissure with demonstrated extravasation of contrast

**Table 2:** Baseline characteristics.

Characteristic	Overall		
	Total	Mean or *N*	SD or %
Age at injection (years)	31	53.4	8.5
Number of levels	31	2.1	0.8
Female sex	31	19	61
Multiple levels injected	31	17	55
Levels injected			
L1-L2	31	0	0
L2-L3	31	3	10
L3-L4	31	8	26
L4-L5	31	25	81
L5-S1	31	17	55
Baseline PROMs			
Current NRS pain	31	5.6	1.6
Best NRS pain	31	4.1	1.9
Worst NRS pain	31	6.9	1.4
FRI score	31	52.5	12.1
SF-36 pain score	31	45.0	13.1
SF-36 physical function score	31	51.8	13.1

FRI: functional rating index; NRS: numerical rating scale; PROM: patient-reported outcome measure; SD: standard deviation; SF-36: 36-item short form.

**Table 3:** Patient-reported outcome measures at 48 weeks postinjection.

PROM	Overall		
	Total	Mean	SD
Current NRS pain	31	3.4	1.4
Best NRS pain	31	3.0	1.9
Worst NRS pain	31	4.6	1.8
FRI score	31	39.2	7.0
SF-36 pain score	31	66.8	18.1
SF-36 physical function score	31	67.7	14.4

FRI: functional rating index; NRS: numerical rating scale; PROM: patient-reported outcome measure; SD: standard deviation; SF-36: 36-item short form.

**Table 4:** Effect of age, sex, and number of levels on success.

Parameter	Failure (*n* = 9)		Success (*n* = 22)		*P* value
	Mean or *N*	SD or %	Mean or *N*	SD or %	
Females (vs. males)	6	66%	13	59%	>0.99
Age at injection (years)	56.2	8.6	52.3	8.4	0.25
Number of levels	2.2	0.7	1.6	0.7	0.03
Multilevels injected	5	55%	2	9%	0.01

SD: standard deviation.

## Data Availability

The data analysed during the current study are not publicly available due to the data being confidential; however, they are available from the corresponding author on reasonable request.
